# Rural Obesity Counseling for Adults Living With Intellectual and Developmental Disabilities Under the Medicaid Program

**DOI:** 10.1016/j.focus.2026.100530

**Published:** 2026-07-15

**Authors:** Yonsu Kim, Ian Choe, Yoohwan Kim, Maryam Tabrizi, Young Sung Kim, Iulia Ioanitoaia-Chaudhry, Maria Teresa Chong, Hanna Namkung, Isabelle Kwak, Ji Won Yoo

**Affiliations:** 1 Department of Healthcare Administration and Policy, School of Public Health, University of Nevada, Las Vegas, Las Vegas, Nevada; 2 Division of Digital Health, OptumHealth, Las Vegas, Nevada; 3 Department of Internal Medicine, Kirk Kerkorian School of Medicine at UNLV, Las Vegas, Nevada; 4 Department of Computer Science, Howard Hughes College of Engineering, University of Nevada, Las Vegas, Nevada; 5 Department of Biomedical Sciences, School of Dental Medicine, University of Nevada, Las Vegas, Las Vegas, Nevada; 6 Department of Anesthesiology and Pain Medicine, Korea University Guro Hospital, Seoul, Republic of Korea; 7 Geriatric Education Center, VA Southern Nevada Health Care, North Las Vegas, Nevada; 8 Department of Family and Community Medicine, Kirk Kerkorian School of Medicine at UNLV, Las Vegas, Nevada; 9 Institute of Human Nutrition, Columbia University Irving Medical Center, New York, New York; 10 The Connection Sphere, Las Vegas, Nevada

**Keywords:** Health disparities, intellectual and developmental disabilities, Medicaid, obesity, rural

## Abstract

**Introduction:**

This study aims to compare the efficiency and outcomes in adults with obesity and intellectual and developmental disabilities in rural areas.

**Methods:**

Utilizing the administrative database of the rural health system and Nevada Medicaid (January 1, 2022 and December 31, 2024), 257 adults with obesity and with or without intellectual and developmental disabilities were identified. A propensity score was applied to match demographics and comorbidity conditions between subjects with and without intellectual and developmental disabilities. Logistic regression analysis was used to identify the factors associated with behavioral counseling for obesity.

**Results:**

Those with intellectual and developmental disabilities were less likely to have behavioral counseling for obesity than the non–intellectual and developmental disability cohort (16.9% vs 30.7%; OR=0.55; 95% CI=0.24, 0.89; *p*=0.03). Being aged 45–64 years, being female, having mental health conditions, and being telehealth users were associated with higher probabilities of receiving behavioral counseling for obesity.

**Conclusions:**

Primary care workforce training is warranted to reduce health disparities by integrating behavioral counseling for obesity among those with special care needs as well as those with limited access to care.

## HIGHLIGHTS

• This study evaluated outcomes for rural adults with obesity and intellectual and developmental disabilities (IDDs).
• Those with IDDs (16.9%) were less likely to receive obesity counseling than those without IDDs (30.7%).
• Age 45–64 years, female, mental health, and telehealth facilitated counseling.

## INTRODUCTION

Residents in rural areas have less access to healthcare services than people in urban areas, tend to be less physically active, and have higher rates of obesity in the U.S.^1^^,^^2^ Obesity remains a major determinant of other cardiometabolic diseases. Social risks, for example, higher food insecurity and poverty rates, in rural areas add up to higher rates of cardiometabolic diseases than in urban populations.^1^^,^^2^ This trend results in critical geographic health disparities in general and in the special needs population, for example, adults living with intellectual and developmental disabilities (IDDs) who are at risk of consuming processed foods and taking antipsychotics or mood stabilizers that alter their metabolisms and eating habits.^3^, ^4^, ^5^ The prevalence of obesity among adults living with IDDs is higher than that in the general population and has been reported to be up to 59%.^5^

In response to this obesity epidemic in the U.S., the Center for Medicare and Medicaid Services started to offer reimbursement for intensive behavioral therapy for obesity in 2012, allowing designated healthcare providers.^6^^,^^7^ Reimbursement rates for the most common Evaluation & Management codes (99213 or 99214) in primary care for equivalent time are at least 3 times higher than the reimbursement amount for behavioral counseling for obesity (G0477, $25.30 in nonfacility; $22.30 in facility in the year 2025).^6^^,^^7^ Healthcare providers, especially primary care providers, decided that they would lose revenue if they only billed for behavioral counseling for obesity. In addition, Medicaid beneficiaries in rural areas are hindered from receiving primary care in a timely manner owing to the limited number of providers and transportation challenges.^8^^,^^9^ Thus, obesity care for adults living in rural areas is critically necessary to explore those with special care needs, individuals with IDDs under the Medicaid payor program.^8^^,^^10^^,^^11^ Telehealth integrating primary care with behavioral health in rural areas may fill the gaps of limited access to care, particularly among individuals with obesity and special care needs.^9^, ^10^, ^11^, ^12^, ^13^ The target population of this study is rural Medicaid beneficiaries, who are the major payor source beneficiaries among adults living in rural areas and with disabilities. The purpose of this study is to examine primary care practice patterns for rural obese Medicaid beneficiaries living with IDDs in rural areas.

## METHODS

### Study Sample

**Figure 1:**
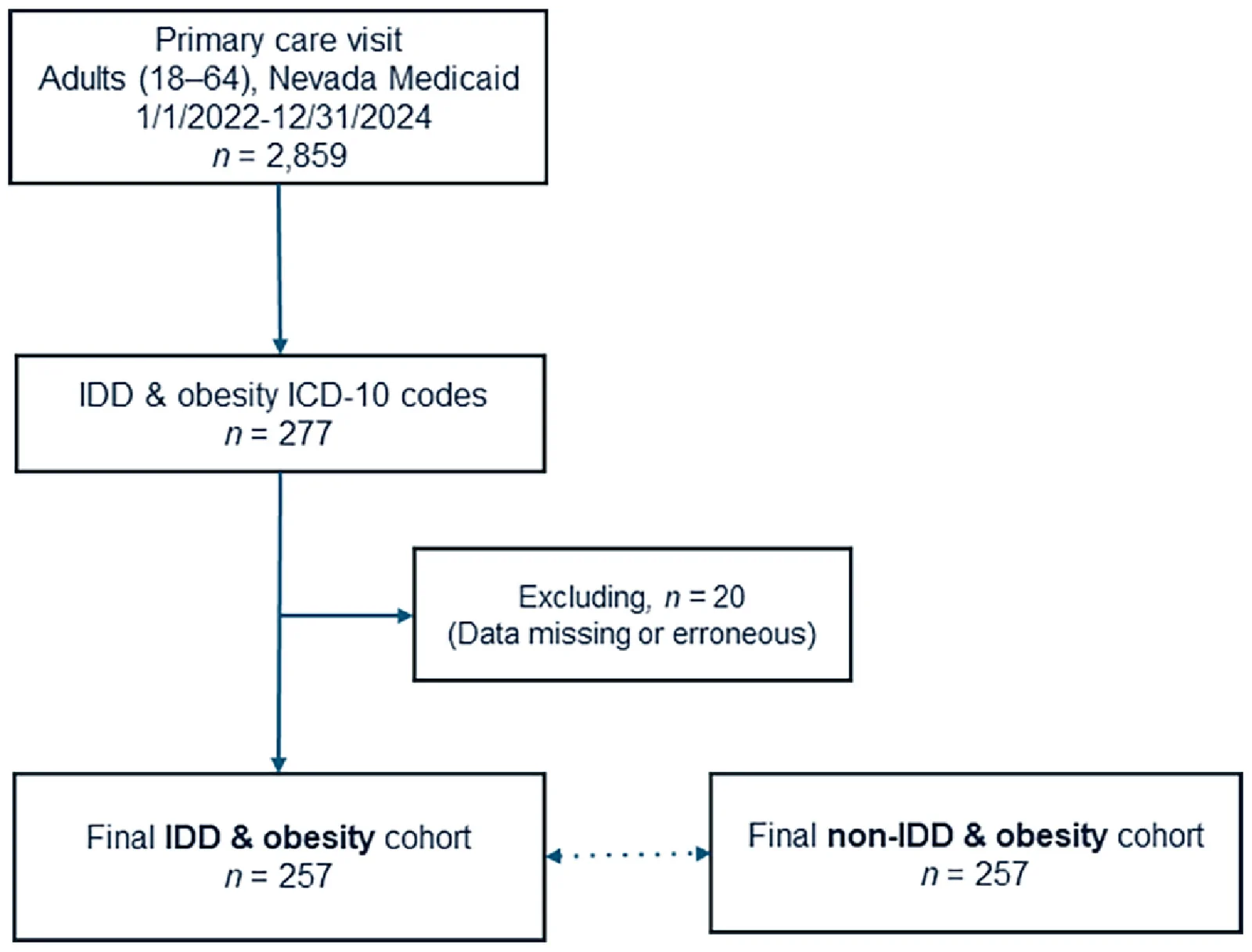
Cohort recruitment flow.

This study utilized the integrated database of safety-net health systems in eastern rural Nevada and the Nevada Medicaid program for analysis. No other healthcare access is available within a 150-mile radius for approximately 100,000 residents. Telehealth has been applied since the coronavirus disease 2019 (COVID-19) pandemic as primary care and for behavioral health. This database is a deidentified, integrated administrative and clinical data platform. Clinical data were extracted from the electronic health record, Meditech (Medical Information Technology, Inc., Westwood, MA), including billing information. Specific diagnostic and procedure codes were used as a query and analysis tool in the integrated database. Figure 1 demonstrates the cohort recruitment flow. A total of 2,859 adults aged 18–64 years received primary care under Nevada Medicaid between January 1, 2022 and December 31, 2024. Of these, 277 individuals were selected using the ICD-10 diagnosis of IDD and obesity. The IDD conditions included Down syndrome, autism spectrum disorder, intellectual disability, and cerebral palsy. Table A.1 (available online) provides the ICD-10 codes for selecting the study cohort. Twenty individuals were excluded because of either missing or erroneous data. Multiple imputation methods using chained equations confirmed that the data were free from the influence of missing or erroneous data for both IDD and non-IDD cohorts.^12^ For the final analysis, 257 individuals with obesity and with IDDs were chosen. Subsequently, a computer-assisted nearest neighbor matching process was carried out by pairing demographics and comorbidity conditions to identify obese and non-IDD individuals (*n*=257) during the same timeframe.^11^ Matching was used only for cohort creation. The IRB at the University of Nevada, Las Vegas exempted review of this study because it was determined to be a quality improvement/evaluation. Therefore, this study was not subject to IRB approval and oversight as human subject research as defined under federal regulations (Number 1613064-1). Informed consent was not obtained owing to the use of a deidentified database.

### Measures

The outcome measurement was behavioral counseling for obesity, which was captured from electronic health record and administrative data using the Current Procedural Terminology and Healthcare Common Procedure Coding System, as depicted in Table A.1 (available online**)**. Demographic information—age, sex, and race/ethnicity—was collected from an administrative database. Race/ethnicity was divided into Whites and others. Comorbid conditions included mental health conditions (either depression, bipolar disorders, schizophrenia/schizoaffective disorders, or attention deficit hyperactive disorders) and Type 2 diabetes. These conditions were common and clinically relevant to individuals with obesity with IDD. Delivery type was defined on the basis of whether telehealth or in-person visits were utilized for primary care.

### Statistical Analysis

Propensity score matching was applied to identify adults with obesity without IDDs who shared similar characteristics with individuals with IDDs. Logistic regression was used to estimate the propensity to have an IDD diagnosis, with demographics (age, sex, race/ethnicity) and comorbidity conditions (mental health conditions and Type 2 diabetes) as independent variables. The IDD versus non-IDD matching ratio was 1:1. For the categorical variables (sex, race/ethnicity, comorbidity conditions), chi-square test was used to confirm no difference between IDD and non-IDD groups. For the outcome measurement (behavioral counseling for obesity), logistic regression analysis was applied to estimate OR of behavioral counseling for obesity for the controlled variables (IDD status, age, sex, race/ethnicity, comorbid conditions, and delivery type). This analysis included an interaction term between IDD and telehealth in the regression model. All statistical analyses were 2 tailed, and *p*-value less than 0.05 was considered statistically significant. SAS (Version 9.4, SAS Institute, Cary, NC) was used for all statistical analyses.

## RESULTS

Of the 257 individuals in the IDD cohort, described in Table 1, the chi-square test revealed that the majority of participants across both cohorts were aged between 18 and 29 years, representing 52.7% (*n*=135) of the IDD and 51.2% (*n*=132) of the non-IDD cohorts. Those aged 30–44 years accounted for 24.3% (*n*=63) and 24.7% (*n*=64) of the IDD and non-IDD cohorts, respectively, whereas those aged 45–64 years comprised 22.1% (*n*=59) and 24.1% (*n*=61) of the IDD and non-IDD cohorts, respectively. Slightly less than 40% of the individuals were female. In terms of race/ethnicity, 87.5% were non-Hispanic Whites, and 12.5% were others. Approximately, 4 in 10 individuals with IDDs had mental health conditions (42.4%), and 1 in 4 IDD cohorts had Type 2 diabetes (25.2%). Behavioral counseling for obesity was less common among the IDD cohorts (16.9%) than among the non-IDD cohorts (30.7%, *p*=0.03).

**Table 1:** Demographic and Clinical Characteristics of Study Cohort Subjects

		Cohorts		
Variables		IDD, *n*=257 % (*n*)	Non-IDD, *n*=257 % (*n*)	*p*-value
Age, years	18–29	52.7% (135)	51.2% (132)	0.96
	30–44	24.3% (63)	24.7% (64)	
	45–64	22.1% (59)	24.1% (61)	
Sex	Male	61.8% (159)	59.6% (153)	0.37
	Female	38.2% (98)	40.4% (104)	
Race/ethnicity	Non-Hispanic Whites	84.1% (216)	86.0% (221)	0.29
	Others	15.9% (41)	14.0% (36)	
Comorbid conditions	Mental health conditions	42.4% (109)	41.0% (105)	0.35
	Type 2 diabetes	25.2% (65)	22.9% (59)	0.42
Outcome measurement	Behavioral counseling for obesity	16.9% (43)	30.7% (79)	0.03

IDD, intellectual developmental disorder.

Table 2 presents the ORs of behavioral counseling for obesity. Individuals with IDDs were less likely to receive behavioral counseling for obesity than non-IDD individuals (OR=0.55, 95% CI=0.24, 0.89, *p*=0.03). Those aged 45–64 years were more likely to have behavioral counseling for obesity than those aged 18–29 years (OR=3.16, 95% CI=1.85, 4.72, *p*<0.001). Female individuals were more likely to receive behavioral counseling for obesity (OR=1.80, 95% CI=1.11, 3.28, *p*=0.03). Telehealth users were more likely to receive behavioral counseling for obesity than those who visited in person (OR=2.28, 95% CI=1.21, 3.47, *p*=0.005). The interaction term between IDD and telehealth visit was not significant (*p*=0.26).

**Table 2:** Factors Associated With Behavioral Counseling for Obesity

Variables	ORs	95% CI		*p*-value
	ref=non-IDD			
IDD	0.55	0.24	0.89	0.03
Age category, years	re=18–29			
30–44	1.12	0.63	1.88	0.36
45–64	3.26	1.85	4.72	<0.001
Sex	ref=male			
Female	1.80	1.11	3.28	0.03
Race/ethnicity	ref=Whites			
Others	0.91	0.48	2.25	0.56
Comorbid conditions	ref=none			
Mental health conditions	2.31	1.27	3.94	0.01
Type 2 diabetes	1.15	0.84	1.59	0.48
Delivery type	ref=in-person visit			
Telehealth visit	2.28	1.21	3.47	0.005

IDD, intellectual developmental disability.

## DISCUSSION

This study examined the primary care practice patterns of obesity counseling for those living with IDD in rural areas. On the basis of the available literature, this study is the first report analyzing primary care practice patterns to serve individuals with special care needs—a combination of obesity, IDD, rural residents, and Medicaid beneficiaries—using billing and administrative information. The main finding of this study was that individuals with IDDs were less likely (55%) to receive behavioral counseling for obesity than non-IDD individuals. In addition, very few (16.9%) individuals with IDDs received behavioral counseling for obesity. These findings raise concerns about the underutilization of obesity care for populations with special care needs in real-world rural communities.

Telehealth users were more than twice as likely to receive behavioral counseling than those making in-person visits. This finding is consistent with the surge in telehealth utilization for obesity counseling since the COVID-19 pandemic, which led to more comprehensive reimbursement parity and flexibility in services modality.^11^^,^^14^^,^^15^ There are opportunities for obesity care through telehealth’s facilitation of behavioral counseling.

Both telehealth and IDD care have not been well known to primary care providers serving this population. Workforce enhancement is prioritized to reduce the above health disparities by training primary care providers on how to communicate with adults with IDDs and their caregivers to manage their diet and exercise lifestyle. Integrating primary care and mental health management was identified by primary care providers in rural areas as being necessary for those with IDD, as reported in other studies.^8^^,^^9^^,^^13^ Including telehealth visit curriculum in graduate medical education is necessary to disseminate telehealth as a standard modality of obesity counseling.

Findings of this study have implications and recommendations for policy in the midst of Medicaid payment shifting from fee-for-service to managed care. The BALANCE (Better Approaches to Lifestyle and Nutrition for Comprehensive hEalth) and Rural Health Transformation programs, which were recently initiated by the Center for Medicare and Medicaid Services, align with the enhancement of access to care for adults living with obesity and in rural areas.^16^^,^^17^ These programs aim at attenuating social risk factors of cardiometabolic diseases in rural areas by supporting lifestyle and nutrition support as well as innovative care delivery in rural areas.^1^^,^^16^^,^^17^ Obesity is a major determinant of other cardiometabolic diseases. As the life expectancy of individuals with IDD has been increasing, the public health burdens of managing obesity among these individuals demand more efficient care delivery, particularly in rural areas.^18^ Because Nevada is a statewide provider shortage state, a telehealth-based obesity counseling model has demonstrated a sustainable and efficient care delivery model, particularly for Medicaid beneficiaries in rural Nevada, where healthcare and community resource utilizations are limited. As one of the workforce training programs, the state medical license authority may enact statewide nutrition continuous medical education programs required for nonnutritionist healthcare providers to provide healthcare services for state residents with obesity, particularly in rural areas and those with special care needs. To successfully take advantage of this policy momentum, hybrid solutions that include both an online telehealth interoperation platform and on-site community engagement programs that promote telehealth and remote monitoring utilizations in rural areas are urgently warranted.

### Limitations

Limitations of this study include the possibility of under-recognizing or incompletely documenting the actual characteristics of study samples in administrative databases. Individuals with overweight (ICD-10 E66.3) were not included in the study samples because of the outcome measurement definition of obesity counseling’s billing feasibility. The lack of detailed clinical information on obesity severity (e.g., BMI class), type of telehealth (e.g., video and telehealth visits), and type of obesity counseling and IDD in the administrative data may contribute to this potential underestimation and lead to selection bias. Differences in the distribution of BMI categories between the IDD and non-IDD groups may partially explain the observed difference in counseling rates. Current research also acknowledges the lack of socioeconomic variables, such as household income, household composition, and caregiving/support arrangements, and this might raise the potential for unmeasured bias. The findings of this study are limited to a single rural geographic area and a single critical access organization with a single payment system population. Further studies with statewide partner systems with different practice characteristics may yield new and different findings.

## CONCLUSIONS

Findings suggest that behavioral counseling was underutilized for adults with obesity living with IDD in rural areas. Primary care workforce training is warranted to reduce health disparities and to advance the care efficiency of adults living with IDDs if telehealth is utilized for primary care delivery for this population.

## CRediT authorship contribution statement

**Yonsu Kim:** Methodology, Software, Formal analysis, Visualization, Writing – original draft, Writing – review & editing. **Ian Choe:** Conceptualization, Writing – original draft, Writing – review & editing. **Yoohwan Kim:** Software, Validation. **Maryam Tabrizi:** Writing – original draft, Writing – review & editing. **Young Sung Kim:** Methodology, Software, Formal analysis. **Iulia Ioanitoaia-Chaudhry:** Conceptualization, Writing – original draft, Writing – review & editing. **Maria Teresa Chong:** Conceptualization, Writing – original draft. **Hanna Namkung:** Visualization, Project administration. **Isabelle Kwak:** Project administration, Visualization. **Ji Won Yoo:** Funding acquisition, Software, Writing – original draft, Writing – review & editing.

## Appendix Supplementary materials

Image, application 1

## Footnotes

Supplementary material associated with this article can be found in the online version at doi:10.1016/j.focus.2026.100530.
