# Supplementary material for: Rural Obesity Counseling for Adults Living With Intellectual and Developmental Disabilities Under the Medicaid Program
Source: AJPM Focus. 2026 Jul 15;5(6):100530. doi: 10.1016/j.focus.2026.100530 (PMC13586564; doi:10.1016/j.focus.2026.100530)
Supplement: Supplementary file 1 [file mmc1.pdf]

Article title: Rural obesity counseling for the adults living with intellectual and developmental disabilities under the Medicaid program
Authors: Yonsu Kim, Ian Choe, Yoohwan Kim, Maryam Tabrizi, Young Sung Kim, Iulia Ioanitoaia-Chaudhry, Maria Teresa Chong, Hanna Namkung, Isabelle Kwak, Ji Won Yoo
Journal name: American Journal of Preventive Medicine Focus

Table A.1. ICD-10 and CPT/HCPCS codes of study cohort and outcome measurement

	Diagnosis	ICD-10 codes
Study cohort	Down syndrome	Q90, Q90.1, Q90.2, Q90.9
	Autism spectrum disorder	F84.0, F84.5, F84.8, F84.9
	Intellectual disability	F70, F71, F72, F73, F78, F79
	Cerebral palsy	G80.0, G80.1, G80.2, G80.3, G80.4, G80.8, G80.9
	Obesity	E66.01, E66.09, E66.1, E66.2, E66.8, E66.9
	Obesity in pregnancy	O99.210, O99.211, O99.212, O99.213, O99.214, O99.215
Mental health conditions	(a) Depression	F32.0, F32.1, F32.2, F32.3, F32.4, F32.5, F32.8, F32.9, F32.A<br>F33.0, F33.1, F33.2, F33.3, F33.4
	(b) Bipolar disorders	F31.0, F31.1, F31.2, F31.3, F31.4, F31.5, F31.6, F31.7, F31.8, F31.9
	(c) Schizophrenia/schizoaffective disorders	F20.0, F20.1, F20.2, F20.3, F20.5, F20.8, F20.9<br>F25.0, F25.1, F25.8, F25.9
	(d) Attention deficit hyperactive disorders	F90.0, F90.1, F90.2, F90.8, F90.9
Comorbid conditions	Type 2 diabetes	E11.0, E11.1, E11.2, E11.2, E11.4, E11.5, E11.6, E11.8, E11.9
		CPT/HCPCS codes
Delivery type	Place of service: other than home, home	2, 12
	Telehealth use	99441, 99442, 99443, 99444, 99421, 99422, 99423, 98790, 98790-2, 98791, 98792, G0071, G2025, G2061, G2061-3, G2062, G2063, G2250-2
Outcome measurement	Behavioral counseling for obesity	G0447

Note: ICD-10, International Classification of Diseases, 10th revision; CPT, Current Procedural Terminology; HCPCS, Healthcare Common Procedure Coding System
